# Cerebrospinal fluid–lymphatic fistula in a child with generalized lymphatic anomaly treated with targeted blood patch — a rare case report and review of the literature

**DOI:** 10.1007/s00381-024-06287-x

**Published:** 2024-01-18

**Authors:** Radek Frič, Ingvild Heier, Mark Züchner, Øivind Gjertsen, Mehran Rezai

**Affiliations:** 1 Department of Neurosurgery, Oslo University Hospital-Rikshospitalet, P.O.Box 4950, N-0424 Oslo, Norway; 2 Department of Pediatric Hematology and Oncology, Oslo University Hospital-Rikshospitalet, Oslo, Norway; 3 Department of Radiology and Nuclear Medicine, Oslo University Hospital, Oslo, Norway

**Keywords:** Lymphatic malformation, Intracranial hypotension, Cerebrospinal fluid leak

## Abstract

Spontaneous intracranial hypotension may result in debilitating postural headaches and severe neurological symptoms due to secondary cerebellar sagging. The most common cause is the cerebrospinal fluid (CSF) leak within the spinal canal. Although previously reported in only a few cases, also paraspinal lymphatic malformations causing vertebral bone destruction may occasionally result in CSF leak to these pathological formations. Here, we present a case of a 9-year-old girl with generalized lymphatic anomaly (GLA) presenting with severe postural headache. Radiological imaging revealed a typical feature of cerebellar sagging. Myelography localized the CSF leakage into vertebral bodies of C7 and Th1, which both were partly involved in pathological paravertebral masses of known lymphatic anomaly, and from there along the right C8 nerve root sleeve into the anomaly. As the C8-nerve root could not be ligated due to the risk of significant neurological injury, we attempted image-guided targeted percutaneous epidural placement of a blood patch directly into the foramen at the affected level. The procedure resulted in obliteration of the fistula and regression of cerebellar sagging, with significant relief of symptoms. Although it is an extremely rare coincidence, patients with paraspinal lymphatic malformations may develop intraspinal CSF leak into these pathological formations. The present case report suggests that besides a direct surgical obliteration of the fistula and sacrificing the nerve root, a targeted percutaneous epidural blood patch may be a possible alternative in the case of a functionally important nerve root.

## Introduction

Spontaneous intracranial hypotension with cerebellar sagging presents often with debilitating postural headaches and may be encountered in adults as well as in children. The most common cause is a cerebrospinal fluid (CSF) leak through the defect in the spinal dural sac, either due to a dural tear, ruptured meningeal diverticula or CSF-venous fistulas [1]. However, there have exceptionally been reported [2–6] also spinal CSF fistulas into paravertebral lymphatic masses in patients harboring lymphatic anomalies like Gorham-Stout disease (GSD), generalized lymphatic anomaly (GLA) or kaposiform lymphangiomatosis (KLA) (Table 1). In these few cases, the patients were treated with epidural and/or transforaminal blood patch, surgical obliteration or liquid embolization, respectively.

Here, we report a case of a pediatric patient with GLA, in whom CSF-lymphatic fistula at the level of the C8 nerve root was successfully managed by image-guided percutaneous epidural placement of a blood patch into the foramen at the affected level.

## Case report

The patient was a 9-year-old girl, who already at the age of 2 years was hospitalized in another country due to pneumonia, pleural empyema and possible chylothorax. Thoracic magnetic resonance imaging (MRI) including lymphangiography at that time had showed bilateral pleural thickening and cysts/fluid accumulations at several thoracic and lumbar levels, in the ribs and the spleen. No osteolytic lesions or cortical defects were present. The diagnosis of GLA was made based on the clinical and radiological findings, but no biopsy of the pathological lymphatic tissue was performed. At 4 years of age, she was also diagnosed with optic neuritis, including the presence of myelin oligodendrocyte glycoprotein (MOG) antibodies. Visual symptoms at that time improved within 1 week, following treatment with systemic steroids. In the following years, she was free of symptoms, and regular radiological follow-up did not show any sign of progression of lymphatic lesions.

Seven years later, in July 2022, she presented with a 4-day history of severe postural headache, clearly increasing in the upright position and accompanied by nausea. On admission, she was tired, nauseous and poorly cooperating. Blurred vision, urinary retention, unsteadiness and dizziness were found on neurological examination.

**Fig. 1:**
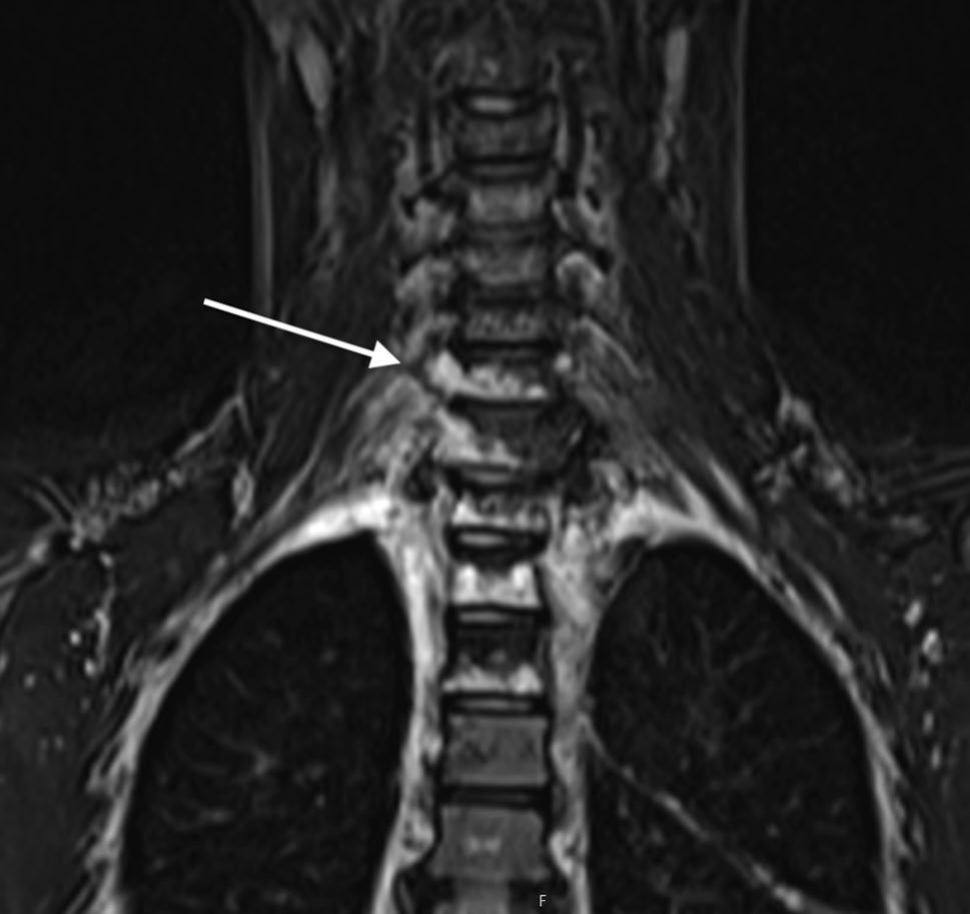
MRI showing the extent of paravertebral lymphatic malformation on the right side, also involving several vertebral bodies at the cervicothoracic junction (white arrow)

**Fig. 2:**
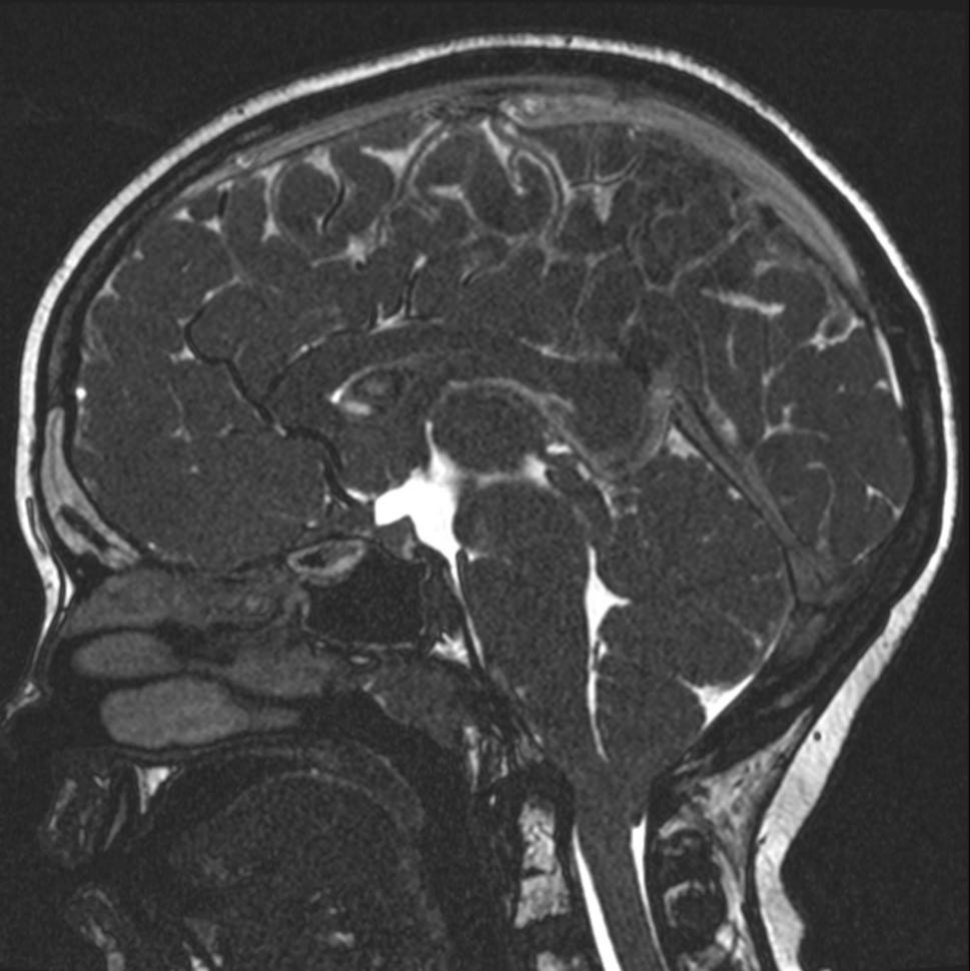
MRI demonstrating cerebellar sagging at the time of clinical presentation

A new MRI showed — along with known lymphatic malformation (Fig. 1) — signs of intracranial hypotension including cerebellar sagging, where the tonsils were located 13 mm under the level of foramen magnum (Fig. 2).

**Fig. 3:**
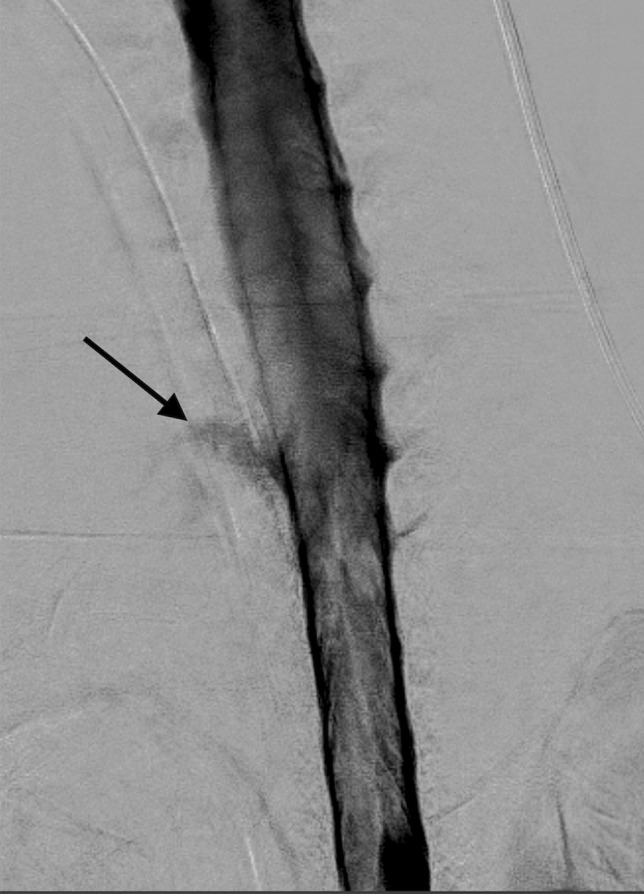
CSF-lymphatic fistula along the right C8 root nerve (black arrow), as revealed on myelography

To localize the level of CSF leak in the spinal canal, we performed spinal myelography with positional maneuvers. A leaking point was identified at the level of the right C8 nerve root sleeve: here, the CSF leaked into vertebral bodies of C7 and Th1, which both were partly involved in pathological paravertebral masses of known lymphatic anomaly, and from there along the nerve into the anomaly (Fig. 3).

**Fig. 4:**
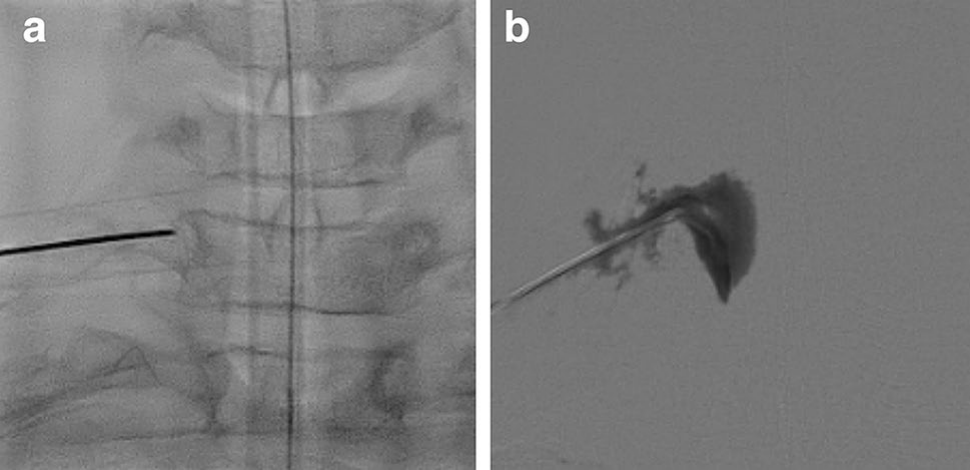
(**a**) Image-guided percutaneous epidural instillation of an autologous blood patch directly into the right foramen C7/Th1; (**b**) from the pedicle, the contrast leaked laterally and inferiorly into paravertebral soft tissue

We did not consider surgical ligation of the C8-root to be a reasonable option, due to the risk of resulting neurological morbidity. Instead, we opted for image-guided targeted percutaneous instillation of an autologous blood patch directly into the foramen C7/Th1. The procedure was successfully performed by the interventional radiologist (M.R.) with a total of 5 ml blood, out of which 2 ml medially in the root canal and right Th1 pedicle; from the pedicle, the contrast leaked laterally and inferiorly in paravertebral soft tissue (Fig. 4).

**Fig. 5:**
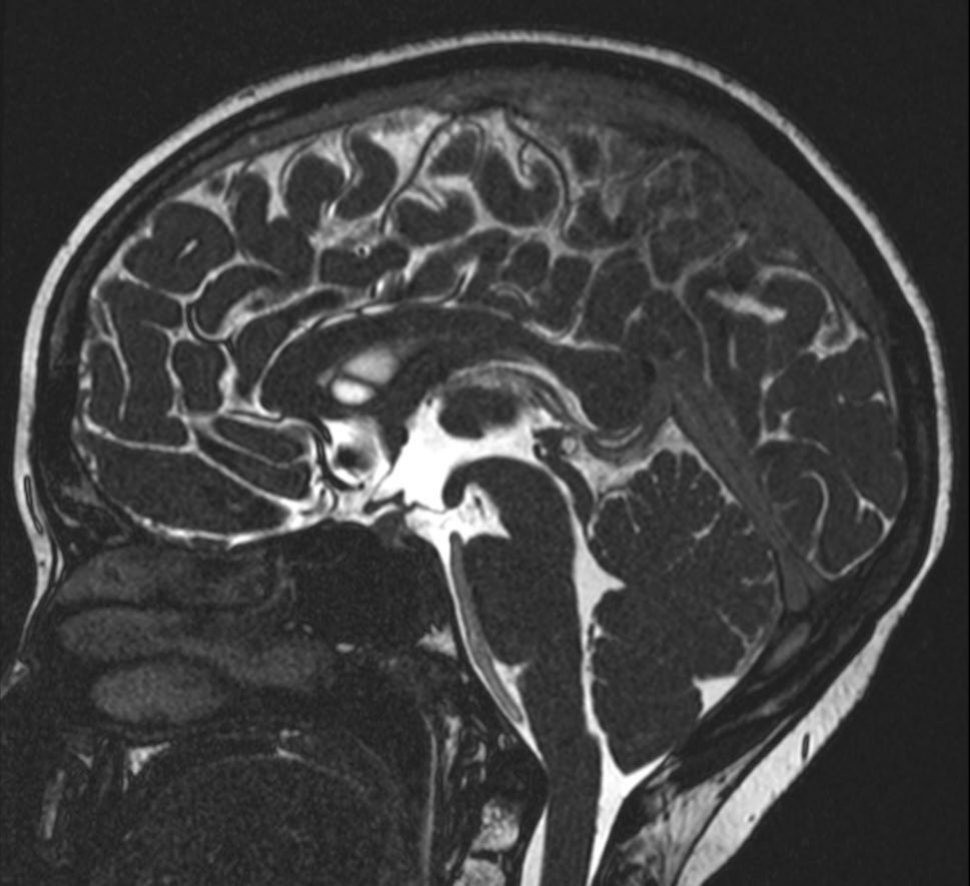
Resolution of cerebellar sagging, as demonstrated on MRI taken 2 weeks after the procedure

Simultaneously, medical treatment directed against GLA with sirolimus was started [7]. The girl recovered very well from her headache. A follow-up MRI taken a few days after the procedure showed a resolution of cerebellar sagging (Fig. 5). However, she developed edema in the cervicothoracic spinal cord with positivity for MOG antibodies, regarded as possible immunological encephalomyelitis. Treatment with systemic steroids was initiated and the symptoms gradually improved.

On the last follow-up controls seven and 15 months after the procedure, respectively, she was found in excellent shape and without any neurological symptoms, except for slight fatigue and polyarticular pain, but also pain in her legs after excessive motion. She would continue the medical treatment as well as radiological follow-up for her lymphatic anomaly. Also, the latest MRI control after 15 months showed regression of signal abnormalities in both the brain and cervical spinal cord, previously possibly associated with intracranial hypotension.

## Discussion

Patients presenting with intracranial hypotension causing cerebellar sagging and severe postural headaches most often have a cerebrospinal fluid (CSF) leak through the defect in the spinal dural sac due to either dural tear, ruptured meningeal diverticula or CSF-venous fistulas [1]. It is also known that patients with lymphatic malformations of cranial bones may develop CSF fistulas [8–10]. However, intraspinal CSF-lymphatic fistulas are extremely rare. After having reviewed the English literature cited in PubMed and cross-checked available references, we identified similar reports from only four patients with GDS and one patient with KLA (Table 1): Adler et al. [2] reported a case of a young child with GDS and long-standing intracranial hypotension, even being operated for secondary Chiari malformation, before CSF leak through the right L4 nerve root sleeve into the expanded L4 and L5 masses was demonstrated. Following repeated attempts on blood patch, CT-guided placement of liquid embolic into the receiving cavity of the fistula was ultimately performed. Suero-Molina et al. [3] presented a case of a 30-year-old male with GDS, where the CSF leak at the level of Th11 was first attempted to be treated by an epidural blood patch, followed by surgical ligation and sealing of the root. Recently, Yokoi et al. [6] reported a case of a child with GDS and CSF leak into osteolytic posterior bony elements Th9-10, treated with direct surgical repair and vertebral fixation. Soderlund et al. [4] presented a case of a child with KLA who developed postural headaches due to a CSF-lymphatic fistula related to a lymphatic malformation associated with the right Th10 root nerve. In this case, the surgical ligation of the CSF-lymphatic fistula was undertaken, resulting in the resolution of the headache. Finally, Xing et al. [5] successfully treated a 20-year-old male with GDS and CSF-fistula at an unspecified level with an epidural blood patch at the level T11-12. In addition, there have been other reports of patients with GDS presenting with cerebellar herniation, which might also possibly be secondary to a CSF-lymphatic fistula [11], similar to the cases mentioned above.

**Table 1:** Reports presenting cases of intraspinal CSF-lymphatic fistulas published to date

**Study**	**Patient**	**Disease**	**Location**	**Treatment**
Adler et al. [2]	F, 7 y	GSD	right L4	1. targeted transforaminal blood patch × 3
				2. liquid embolization of the receiving cavity
Suero-Molina et al. [3]	M, 30 y	GSD	Th11	1. epidural blood patch
				2. surgical ligation and sealing
Soderlund et al. [4]	F, 9 y	KLA	right Th10	1. targeted transforaminal epidural blood patch
				2. surgical ligation
Yokoi et al. [6]	F, 14 y	GDS	Th9-10	direct surgical repair and fixation Th6-11
Xing et al. [5]	M, 20 y	GDS	Th-L?	epidural blood patch T11-12
Present case	F, 9 y	GLA	right C8	targeted blood patch into the C8 nerve root sleeve

*GLA*, generalized lymphatic anomaly; *GSD*, Gorham-Stout disease; *KLA*, kaposiform lymphangiomatosis

From this overview, it appears that standard “untargeted” epidural blood patch often fails to treat this sort of CSF fistula. Surgical ligation of the affected nerve root is effective, but not justified in the case of functionally important roots at the cervical or lumbar level. In our case, we therefore decided to place the epidural blood patch directly into the foramen at the affected level, with satisfactory clinical and radiological results.

Localizing the CSF fistula, i.e. the point of leakage outside the dura sac, may be challenging and not always successful. In our case, we benefited from positioning the patient in a lateral decubitus position, as described previously [12–14].

## Conclusion

Although it is an extremely rare coincidence, patients with paraspinal lymphatic malformations may develop intraspinal CSF leak into these pathological formations. The present case report suggests that besides a direct surgical obliteration of the fistula and sacrificing the nerve root, a targeted percutaneous epidural blood patch may be a possible alternative in the case of a functionally important nerve root.

## Data Availability

The data supporting the findings of this study are available from the corresponding author upon reasonable request.
